# Prescription rates of adrenaline auto-injectors for children in UK general practice: a retrospective cohort study

**DOI:** 10.3399/bjgp17X689917

**Published:** 2017-03-14

**Authors:** Lavanya Diwakar, Carole Cummins, Ronan Ryan, Tom Marshall, Tracy Roberts

**Affiliations:** Queen Elizabeth Hospital, Birmingham, and research fellow in health economics, University of Birmingham, Birmingham.; Institute of Applied Health Research, College of Medical and Dental Sciences, University of Birmingham, Birmingham.; Institute of Applied Health Research, College of Medical and Dental Sciences, University of Birmingham; and research fellow in research & development, Medical Innovation Research and Development Unit, Heart of England NHS Foundation Trust, Birmingham.; Institute of Applied Health Research, College of Medical and Dental Sciences, University of Birmingham, Birmingham.; Institute of Applied Health Research, College of Medical and Dental Sciences, University of Birmingham, Birmingham.

**Keywords:** allergy, adrenaline, anaphylaxis, database, general practice, prescriptions, primary health care

## Abstract

**Background:**

Adrenaline auto-injectors (AAI) should be provided to individuals considered to be at high risk of anaphylaxis. There is some evidence that the rate of AAI prescription is increasing, but the true extent has not been previously quantified.

**Aim:**

To estimate the trends in annual GP-issued prescriptions for AAI among UK children between 2000 and 2012.

**Design and setting:**

Retrospective cohort study using data from primary care practices that contributed to The Health Improvement Network (THIN) database.

**Method:**

Children and young people aged between 0–17 years of age with a prescription for AAIs were identified, and annual AAI device prescription rates were estimated using Stata (version 12).

**Results:**

A total of 1.06 million UK children were identified, providing 5.1 million person years of follow-up data. Overall, 23 837 children were deemed high risk by their GPs, and were prescribed 98 737 AAI devices. This equates to 4.67 children (95% confidence interval [CI] = 4.66 to 4.69), and 19.4 (95% CI = 19.2 to 19.5) devices per 1000 person years. Between 2000 and 2012, there has been a 355% increase in the number of children prescribed devices, and a 506% increase in the total number of AAI devices prescribed per 1000 person years in the UK. The number of devices issued per high-risk child during this period has also increased by 33%.

**Conclusion:**

The number of children being prescribed AAI devices and the number of devices being prescribed in UK primary care between 2000 and 2012 has significantly increased. A discussion to promote rational prescribing of AAIs in the NHS is needed.

## INTRODUCTION

Anaphylaxis is a ‘serious allergic event that is rapid in onset and may cause death’.^1^ Pre-filled adrenaline auto-injectors (AAI) are provided to individuals at high risk of anaphylaxis for emergency self-administration, to prevent worsening of early symptoms of anaphylaxis.^2^

Since their introduction into the UK in March 1996,^2^ prescription rates of these devices in the community has increased exponentially.^3^ The current UK and European paediatric guidelines suggest that a child with minor reactions to peanuts or tree nuts could be prescribed an AAI.^4^^,^^5^ Others argue that the devices should only be prescribed to children who have experienced moderate or severe allergic reactions.^6^ The British Society of Allergy and Clinical Immunology (BSACI) has also laid down guidance for the prescription of these devices.^7^ However, it is well recognised that reaction history to an allergen does not predict the severity of subsequent reactions.^8^^,^^9^ A recent survey of UK physicians showed that there was a great deal of heterogeneity in the prescription of these devices.^10^

Although previous publications from England and Australia have documented the increase in the number of AAIs being prescribed to children in the community,^3^^,^^11^ the true extent of this in the UK has not yet been explored.

## METHOD

The authors used data from The Health Improvement Network (THIN). The database currently includes information from 587 primary care practices across the UK, covering more than 12 million patients (about 3.6 million active patients), who represent about 6% of the entire UK population.^12^ These data are representative of the UK population by age, sex, medical conditions, and death rates adjusted for demographics and social deprivation.^13^ Participating practices use Vision software to maintain patient records and issue prescriptions. Prescription data from THIN have been previously validated for pharmacoepidemiological research.^14^

### Study population

All children and young people aged 0–17 years registered for a minimum of 1 year between 1 January 2000 and 31 December 2012 in primary care practices contributing to the THIN database were included in the analysis. The children contributed to the dataset from the time of their registration with the practice until the earliest of their 18th birthday, transfer to another surgery, death, or last data collection from the practice.

**How this fits in**

The rate of anaphylaxis in England is increasing, and the number of adrenaline auto-injectors (AAIs) dispensed in England between 2000 and 2012 has also increased. This study demonstrates that more children are being prescribed AAI devices within the UK by their GP, and that the number of devices being prescribed per child is also increasing. The annual costs to the NHS are significant, and a discussion regarding rational prescribing of AAI devices in children is warranted.

### Statistical analysis

The authors identified AAI devices based on their generic names (Table 1) using database specific code lists. All the children contributing to the database were considered at risk and constituted the denominator. It was presumed that children who had had an AAI prescribed were considered to be at risk of anaphylaxis by their GPs and they were designated as high risk for the purposes of the study.

**Table 1.** Adrenaline devices dispensed for use in children and young people aged 0–17 years in the UK

**Generic name**	**% (of total)^a^**	**Age ≤5 years % (of total)^a^**	**Age ≥6 years % (of total)^a^**
**Junior devices (dispense 150 µg)**			
Adrenaline (base) 150 µg/0.15 ml (1 in 1000) solution for injection pre-filled disposable devices	2.48	3.95	1.91
Adrenaline (base) 150 µg/0.3 ml (1 in 2000) solution for injection pre-filled disposable devices	64.56	95.34	52.54

**Adult devices (dispense 300 µg)**			
Adrenaline (base) 300 µg/0.3 ml (1 in 1000) solution for injection pre-filled disposable devices	32.93	0.7	45.49

**Higher dosage devices (dispense 500 µg)**			
Adrenaline (base) 500 µg/0.3 ml (1 in 600) solution for injection pre-filled disposable devices	0.03	0	0.04

**Adrenaline inhalers**			
Adrenaline acid tartrate 280 µg/inhalation inhaler	0.01	0.01	0.01

a There are no figures presented for totals as these are cumulative over 12 years.

The main outcomes of interest were the temporal trends in:

- the number of high-risk children per 1000 UK children (defined as any child who was issued with a prescription for AAI);
- the number of AAI devices issued per 1000 UK children between the years 2000 and 2012; and
- the mean number of devices issued per high-risk child per year.

Evidence for indication for AAI prescription was looked for by searching Read Codes for common food allergies, venom allergy, and some codes for anaphylaxis.^7^ (Box 1)

Box 1. **Read Codes used within the study**

**Table:** 

**Read Code**	**Description**	**Subgroup**
	**Food allergy**	
SN58000	Egg allergy	Egg allergy
SN58 100	Egg protein allergy	Egg allergy
SN58 200	Peanut allergy	Nut allergy
SN58 300	Nut allergy	Nut allergy
SN58 500	Fish allergy	Seafood allergy
SN58 600	Seafood allergy	Seafood allergy
SN58 700	Shellfish allergy	Seafood allergy

	**Anaphylaxis and angioedema**	
SN50.00	Anaphylactic shock	Anaphylactic shock
SN50 000	Anaphylactic shock due to adverse food reaction	Anaphylactic shock

	**Venom allergy**	
SN59.00	Allergic reaction to venom	Venom allergy
SN59 000	Allergic reaction to bee sting	Venom allergy
SN59 100	Allergic reaction to insect bite	Venom allergy
SN59 200	Allergic reaction to wasp sting	Venom allergy
SN59 300	Anaphylactic shock due to bee sting	Venom allergy
SN59 400	Anaphylactic shock due to wasp sting	Venom allergy

Analysis was carried out using Stata (version 12) and graphs were produced using Microsoft Excel 2010.

## RESULTS

A total of 1.06 million UK children <18 years of age were registered between 2000 and 2012, providing 5.1 million person years of follow-up data. Overall, 23 837 children were deemed high risk by their GPs and were prescribed a total of 98 737 AAI devices during the study period. This equates to 4.67 high-risk children (95% confidence interval [CI] = 4.66 to 4.69), and 19.4 (95% CI = 19.2 to 19.5) devices per 1000 person years. About 50% of these children (12 000) were noted as having egg, nut, seafood, insect venom allergy, or previous anaphylaxis by their physicians.

**Figure 1.**
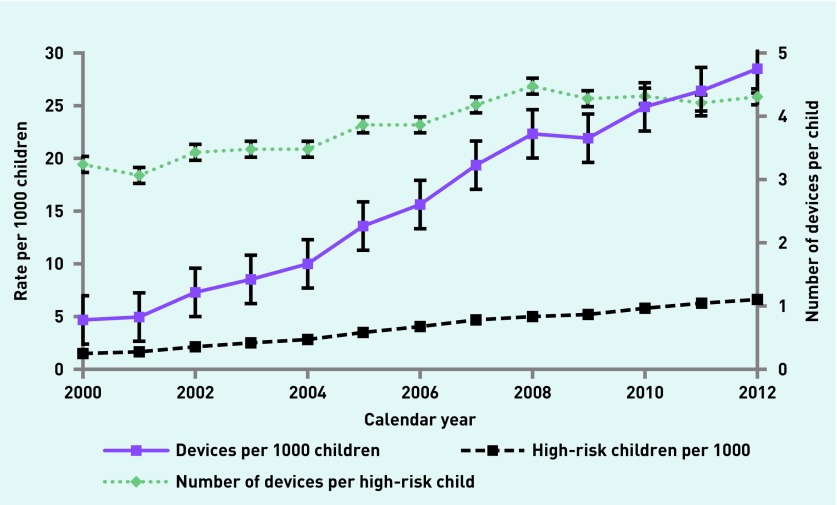
***Trends in proportion of children with AAI devices and the overall rate of AAI device availability in the UK by year.***

The data show a 355% increase in the proportion of children in the community issued with AAI devices, a 506% increase in the proportion of AAI devices prescribed in the community, and a 33% increase in the number of devices issued per high-risk child between the years 2000 and 2012 (Figure 1 and Table 2). The number of devices prescribed per child per year varied significantly, and ranged from 0 to 40. Regression analysis revealed that all these trends are statistically significant at *P*<0.0001.

**Table 2.** Trends in adrenaline autoinjector prescriptions and use between 2000 and 2012

**Year**	**AAI prescriptions issued,^a^ rate**	**Devices in the community,^a^ rate (95% CI)**	**Devices per child, rate (95% CI)**	**Devices in the community for children ≤1 year^a^, rate (95% CI)**
2000	1.45	4.67 (4.22 to 5.13)	3.23 (2.92 to 3.54)	0.38 (0.18 to 0.59)
2001	1.60	4.89 (4.53 to 5.25)	3.05 (2.82 to 3.27)	0.91 (0.65 to 1.16)
2002	2.13	7.29 (6.89 to 7.71)	3.42 (3.22 to 3.62)	0.56 (0.4 to 0.72)
2003	2.47	8.57 (8.14 to 8.99)	3.47 (3.72 to 3.98)	0.51 (0.42 to 0.6)
2004	2.86	9.94 (9.5 to 10.37)	3.48 (3.32 to 3.63)	0.82 (0.61 to 1.03)
2005	3.51	13.49 (13.03 to 13.96)	3.85 (3.72 to 3.98)	1.46 (1.09 to 1.84)
2006	4.04	15.55 (15.08 to 16.02)	3.85 (3.73 to 3.97)	1.05 (0.85 to 1.24)
2007	4.62	19.22 (18.68 to 19.76)	4.16 (4.04 to 4.28)	1.58 (1.31 to 1.85)
2008	5.01	22.28 (21.73 to 22.83)	4.45 (4.34 to 4.56)	0.99 (0.83 to 1.16)
2009	5.14	21.87 (21.35 to 22.38)	4.26 (4.16 to 4.36)	1.77 (1.34 to 2.21)
2010	5.73	24.72 (24.17 to 25.27)	4.31 (4.22 to 4.41)	1.66 (1.44 to 1.88)
2011	6.28	26.32 (25.79 to 26.85)	4.19 (4.11 to 4.27)	1.62 (1.35 to 1.88)
2012	6.59	28.30 (27.75 to 28.86)	4.30 (4.21 to 4.38)	2.55 (2.2 to 2.91)

a Rate per 1000 children. AAI = adrenaline autoinjector..

**Figure 2.**
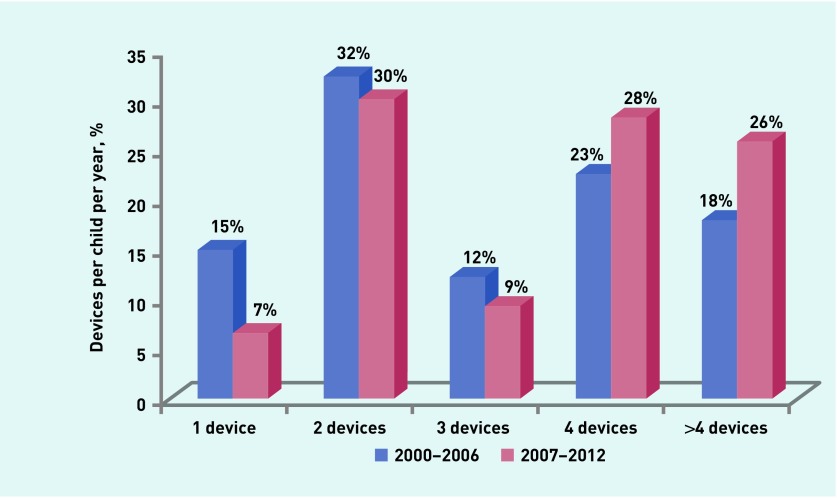
***Proportion of children receiving one to four or more adrenaline auto-injectors (AAI) devices in the years 2000 to 2012.***

In addition, 31% of the high-risk children received two AAI devices, and 27% received four devices per year. A few children (8%) received only one, and 24% received more than four devices per year. On average, each high-risk child received 3.84 devices per year during the study period. When the data were arbitrarily divided into two groups (2000–2006 and 2007–2012), the authors found that the proportion of high-risk children receiving four or more devices is increasing (41% in 2000–2006 versus 54% in 2007–2012) (Figure 2). The types of devices prescribed commonly are shown in Table 1. Almost all of the children ≤5 years of age were prescribed a ‘junior’ device (150 μg adrenaline per dose), whereas just over half of those ≥6 years (52.5%) had a junior device.

The average cost of an AAI device was £25.80 (95% CI = £24.7 to £26.9).^15^ Given that the THIN database represents 6% of the UK population, the authors estimate that the annual expenditure on AAI devices for children in the UK was approximately £6.89 million in 2012.

## DISCUSSION

### Summary

The data show a sharp increase in both the proportion of children prescribed AAI devices and the number of AAI devices prescribed for early management of anaphylaxis between the years 2000 and 2012 in the UK. Given that only half of the ‘high-risk’ children were noted as having a previous relevant allergy or anaphylaxis diagnosis, the rationale for prescribing is, in many cases, unclear.

The authors also found a considerable variation in the number of devices prescribed to children in the UK. One-quarter of all high-risk children receive more than four devices in a year. More than 2% receive >10 devices per year. Of potential concern is the significant increase in the number of devices being prescribed to children ≤1 year old (Table 2), given that AAI devices were, until recently, unlicensed for use in children who weigh <15 kg.^8^^,^^16^ Recent changes to licensing indications for Epipen^®^, however, allow for a junior dose to be prescribed to children who weigh between 7.5 and 25 kg.^17^

The rate of admissions into hospitals in England for anaphylaxis has increased over this period, although mortality rates have decreased.^3^ It is not clear whether these reductions in mortality are directly related to the increase in AAI prescription. Assuming that the admission rates into UK hospitals for anaphylaxis was seven per 100 000 in 2012,^3^ the authors estimate that approximately 400 devices were prescribed per child admitted into hospital in that year. The benefits and risks of such prescribing are not clear. The current annual expenditure on AAI devices for children in the UK is approximately £7 million. Given that these devices have a limited shelf-life and need to be renewed regularly, the costs could escalate considerably if the current trend continues.

### Strengths and limitations

This study is the first to provide individual level, longitudinal data on the availability of AAI among children in the UK. The authors used the THIN database, a large primary care dataset that has been shown to be reliable and representative of the UK population.^13^ Although there are some concerns regarding the quality of data and coding in healthcare datasets in general,^18^ it is accepted that they provide a credible snapshot of healthcare demand and provision across the country.^19^ Primary care practices use their clinical system to issue prescriptions; prescribing data are, therefore, likely to be reliable and complete. Since the majority of adrenaline devices in the UK are obtained using prescriptions from GPs, this analysis is likely to represent the true availability of these devices in the community.

Data from THIN only identify individuals who actively consult their GP. Children who have allergies but do not consult their GP will not be represented in the database. A further limitation of the study is the lack of comprehensive data regarding the indications for AAI prescription, the reasons for repeat prescriptions, and the number of episodes of anaphylaxis/serious allergy experienced by an individual. It was also not possible for the authors to ascertain if all of the prescriptions were dispensed. The dataset only included data until 2012. However, the authors do not expect that more recent data will show significant deviations from the trends demonstrated.

### Comparison with existing literature

The increase in the rate of prescription of AAI devices in England has been reported by others.^3^^,^^20^ However, this is the first report that uses primary care data to provide a detailed analysis of the current primary care prescribing practices for AAI to UK children. The authors are able to demonstrate an increase in the number of children receiving these devices, as well as in the number of devices received per child.

### Implications for practice and research

This study clearly demonstrates that there has been a significant increase in the number of children being prescribed AAI devices in the UK. In addition, there has been an increase in the average number of devices being prescribed per child in the community. This places a significant financial burden on the NHS.

The role of AAI in the management of anaphylaxis is not very clear. Some suggest that it should be administered early in the onset of anaphylaxis, and such use can indeed reduce the need for adrenaline in hospital and subsequent hospital admission.^21^ However, fatalities occur despite early and appropriate use of AAI.^8^ A 2014 review by the Medicines and Healthcare Products Regulatory Agency (MHRA) suggested that the currently marketed AAI devices may not provide a sufficient intramuscular dose of adrenaline in most individuals, increasing the uncertainty around their efficacy in emergencies.^2^ Recent guidance published by the BSACI suggested that only one AAI should be issued per individual, although children may be issued with two (one for home and one for school). Prescribing two devices can be considered in certain situations (for example, in the case of a patient who is obese, remoteness from medical help, or a history of previous anaphylaxis needing two AAI injections).^7^

Although there are some arguments that AAI devices provide reassurance to parents and children, others have shown that carrying these devices is sometimes detrimental to health-related quality of life (HRQoL).^22^ There are no clear guidelines on the optimal number of devices per child. The MHRA has recommended that a second device should be carried by every patient as a backup.^2^ Although a recent survey suggested that most UK clinicians advise that patients carry two AAIs with them at all times,^10^ this analysis suggests that most children are prescribed more than two devices per year.

The short shelf life of the devices at the time of purchase may result in multiple prescriptions in a given year. Parents or carers may request additional devices to hold with childminders or other family members, in addition to those at home and school. In general, there are no data to suggest that provision of extra (beyond two) devices can be life-saving or provide reassurance.^23^ In order to be effective, devices should be prescribed alongside appropriate training and support in their use. Unfortunately, this is often lacking.^8^ In addition, it is difficult to ensure that individuals always carry AAIs with them, that their devices are in date, and that their training is updated regularly to ensure correct administration of the device in an emergency.^8^

Hence there is a need for a robust discussion on the rational prescribing of AAI devices. Indications for AAI prescription among children and on the optimal number of devices that can be issued per child need to be clarified. This will not only have implications for the wellbeing of patients at risk of anaphylaxis, but can also be cost saving to the NHS.
